# Transient Horner’s Syndrome Following CT-Guided C7 Nerve Root Block—A Case Report

**DOI:** 10.1007/s00062-024-01453-9

**Published:** 2024-08-30

**Authors:** Leonhard Mann, Valentina Correa Martelo, Elke Hattingen, Christophe T. Arendt

**Affiliations:** Institute of Neuroradiology, Goethe-University Frankfurt, Schleusenweg 2–16, 60528 Frankfurt/Main, Germany

## Introduction

Selective nerve root block (SNRB) is an effective method for both diagnosing and treating radicular pain syndrome, helping to avoid surgery in over 50% of cases [1]. The aim of SNRB is to infiltrate the nerve root precisely within or close to the intervertebral foramen with local anesthetics and, in most cases, corticosteroids. It can be performed using sonographic, fluoroscopic, or computed tomographic (CT) guidance. Horner’s syndrome is characterized by the typical triad ptosis, miosis, and facial anhidrosis [2]. It can result from an impairment of the oculosympathetic pathway consisting of three neurons: the first neuron originates in the hypothalamus, descending through the hypothalamospinal tract to C8/T1 level, where it synapses with the second neuron in the intermediolateral cell column of the spinal cord. The second neuron leaves the spinal cord with the ventral rami of T1 and enters the sympathetic trunk at the cervicothoracic ganglion (“ganglion stellatum”). It runs with the sympathetic trunk directly next to the vertebra bodies to the superior cervical ganglion, where it synapses with the third neuron. The third neuron leaves the paravertebral region and ascends with the internal carotid artery to the cranium, innervating the dilator pupillae and tarsal muscle [3]. Although typically caused by tumors or carotid dissection, Horner’s syndrome is also reported as a rare complication of neuraxial blockade [4].

## Clinical Case

**Fig. 1:**
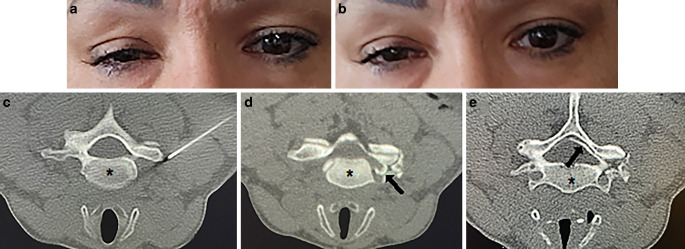
Right-sided ptosis observed two (**a**) and in somewhat lesser extent six (**b**) hours after selective C7 nerve. root. block. Tip of the cannula positioned under computed tomographic guidance at the outer confines of the right-sided neuroforamen C6/C7 to perform selective C7 nerve root block (**c**). Post-procedure control showed distribution of the contrasted mix of local anesthetic and non-crystalloid corticosteroid alongside the C7 root and into the transverse foramen (*arrow* in **d**), as well as into the epidural space (*arrow* in **e**). Note the reversal sides in the images due to the patient being in the prone position. The *Asterisk* * marks vertebra C6

A 42-year-old female was referred to our hospital due to chronic pain in the lower cervical spine radiating into the right arm, accompanied by intermittent hypoesthesia of this arm extending to the digits 2 through 4. She reported worsening symptoms over the last weeks and subjective arm weakness. Non-steroidal anti-inflammatory drugs provided only limited pain reduction. Neurological examination showed no deficits, particularly no paresis of the right arm or hand. Magnetic resonance imaging of the cervical spine showed a disc protrusion at C6/7 level, narrowing the right foraminal zone and affecting the C7 root. Neurosurgeons recommended a selective C7 nerve root block (SNRB) at our Department of Neuroradiology, which we performed per our standard procedure. After a spiral CT to plan the trajectory, the entry site was disinfected and anesthetized. A 10-cm long 20G-needle was advanced towards the posterior aspect of the right-sided neural foramen C6/C7 under CT-guidance (Fig. 1). After verifying correct placement, a mix of mepivacain, dexamethasone, and iodinated contrast agent was administered carefully. A control scan showed contrast distribution alongside the C7 root, into the epidural space, and transverse foramen. After 15 min, we discharged the patient without any complications. Later, she called and reported a right-sided drooping eyelid some minutes after leaving our institute, worsening within the next hour (Fig. 1). No further complaints (i.e., miosis, facial anhidrosis, or symptoms of a stroke) were expressed. We suspected Horner’s syndrome attributed to the intervention. During follow up, she reported that the ptosis decreased within the next hours and completely resolved by the next day. The radicular pain was significantly relieved after SNRB.

## Discussion

We present a rare case of transient Horner’s syndrome following CT-guided C7 SNRB. The control scan showed a distribution of the contrasted medication (local anesthetic and corticosteroid) alongside the C7 root, into the epidural space, and transverse foramen. Anatomically, the most vulnerable location for oculosympathetic pathway affection during SNRB is the second neuron’s exit of the spinal tract at T1 and its course upwards beside the vertebral bodies to the superior cervical ganglion. In the reported case, the contrast pattern suggests involvement of the extraspinal second neuron around the middle cervical ganglion at level C6 [5]. Additionally, medication distribution in the epidural space may have affected the second neuron alongside the ventral rami of T1 nerve root exiting the spinal cord. Several authors reported Horner’s syndrome from epidural anesthesia during labor, assuming a cranial distribution of local anesthetics along the epidural space in supine position [6, 7]. However, literature on Horner’s syndrome following SNRB is scarce. A 2009 study on 802 fluoroscopic-guided SNRB documented six cases of transient Horner’s syndromes, with four following C7 and two following C6 SNRB [8]. Additionally, there are occasional reports of persistent Horner’s syndrome after fluoroscopic-guided SNRB [9]. To date, there is no documented case of this condition following CT-guided SNRB. We performed SNRB with CT guidance for a more precise cannula positioning and chose dorsal approach to minimize injury risk to vulnerable structures [10]. The rapid regression of symptoms in our case suggests nerve involvement by the local anesthetic. This case emphasizes the importance for interventionalists to carefully consider the risk of transient or permanent damage to the second neuron of the oculosympathetic pathway following lower cervical spine SNRB. Although Horner’s syndrome is a documented, rare complication of fluoroscopic-guided SNRB, to our best knowledge, there were no other reports following the CT-guided intervention. Besides known complications of SNRB, such as vessel or nerve injury, infection and allergic reaction [8], physicians should be mindful of Horner’s syndrome as a potential complication irrespective of technique, and consider this during patient informed consent.

## Data Availability

The data of this study are available from the corresponding author on reasonable request.

## Abbreviations

CT: Computed tomography
SNRB: Selective nerve root block
