# Correction to: Transient Horner’s Syndrome Following CT-Guided C7 Nerve Root Block—A Case Report

**DOI:** 10.1007/s00062-026-01683-z

**Published:** 2026-06-09

**Authors:** Leonhard Mann, Valentina Correa Martelo, Elke Hattingen, Christophe T. Arendt

**Affiliations:** Institute of Neuroradiology, Goethe-University Frankfurt, Schleusenweg 2–16, 60528 Frankfurt/Main, Germany

**Correction to:**

**Clin Neuroradiol 2024**

https://doi.org/10.1007/s00062-024-01453-9

The publication of this article unfortunately contained mistakes. The name of one of the authors was not correct. The corrected name is given below.

Incomplete name:

Valentina Correa

Correct name:

Valentina Correa Martelo

The original article has been corrected.

## Data Availability

All data supporting the findings of this work are included in the article.

